# Analysis of HLA DR, HLA DQ, C4A, FcγRIIa, FcγRIIIa, MBL, and IL-1Ra allelic variants in Caucasian systemic lupus erythematosus patients suggests an effect of the combined FcγRIIa R/R and IL-1Ra 2/2 genotypes on disease susceptibility

**DOI:** 10.1186/ar1224

**Published:** 2004-09-23

**Authors:** Andreas Jönsen, Anders A Bengtsson, Gunnar Sturfelt, Lennart Truedsson

**Affiliations:** 1 Department of Rheumatology, Lund University Hospital, Lund, Sweden; 2 Department of Laboratory Medicine, Section of Microbiology, Immunology and Glycobiology, Lund University, Lund, Sweden

**Keywords:** Fcγ receptor, HLA, interleukin-1 receptor antagonist, mannan-binding lectin, systemic lupus erythematosus

## Abstract

Dysfunction in various parts of immune defence, such as immune response, immune complex clearance, and inflammation, has an impact on pathogenesis in systemic lupus erythematosus (SLE). We hypothesised that combinations of common variants of genes involved in these immune functions are associated with susceptibility to SLE. The following variants were analysed: HLA DR3, HLA DQ2, C4AQ0, Fcγ receptor IIa (FcγRIIa) genotype R/R, Fcγ receptor IIIa (FcRγIIIa) genotype F/F, mannan-binding lectin (MBL) genotype conferring a low serum concentration of MBL (MBL-low), and interleukin-1 receptor antagonist (IL-1Ra) genotype 2/2. Polymorphisms were analysed in 143 Caucasian patients with SLE and 200 healthy controls. HLA DR3 in SLE patients was in 90% part of the haplotype HLA DR3-DQ2-C4AQ0, which was strongly associated with SLE (odds ratio [OR] 2.8, 95% CI 1.7–4.5). Analysis of combinations of gene variants revealed that the strong association with SLE for HLA DR3-DQ2-C4AQ0 remained after combination with FcγRIIa R/R, FcγRIIIa F/F, and MBL-low (OR>2). Furthermore, the combination of the FcγRIIa R/R and IL-1Ra 2/2 genotypes yielded a strong correlation with SLE (OR 11.8, 95% CI 1.5–95.4). This study demonstrates that certain combinations of gene variants may increase susceptibility to SLE, suggesting this approach for future studies. It also confirms earlier findings regarding the HLA DR3-DQ2-C4AQ0 haplotype.

## Introduction

The genetic contribution to the aetiology of systemic lupus erythematosus (SLE) is high, as is indicated by familial aggregation and a higher concordance rate in monozygotic than dizygotic twins [1]. The major histocompatibility complex (MHC) haplotype HLA DR3-DQ2-C4AQ0 is strongly associated with SLE in Caucasians [2,3]. The IgG Fc receptors appear to be important in the pathogenesis of SLE, as recently reviewed by Salmon and Pricop [4]. With the allelic variant of R (arginine) instead of H (histidine) on amino acid position 131, the ability of Fcγ receptor IIa (FcγRIIa) to bind IgG_2 _is diminished [5]. Similarly, an amino acid substitution in position 158 (phenylalanine [F] instead of valine [V]) in the Fcγ receptor IIIa (FcγRIIIa) reduces the IgG_1_-, IgG_3_-, and IgG_4_-binding capacity of the receptor [6]. These variants can result in suboptimal clearance of immune complexes from the circulation, which might contribute to the pathogenesis of immune-complex-mediated manifestations [7].

Mannan-binding lectin (MBL) is structurally similar to C1q and has the ability to activate the complement cascade through the lectin pathway. Point mutations are found in the structural gene that affect the MBL serum concentration and the stability of MBL complex formation required for efficient complement activation [8]. In the promoter regions, there are two polymorphisms that influence serum concentration, with LX conferring the lowest MBL level, LY a medium level, and HY the highest [8-11]. MBL variant alleles have been suggested as a minor risk factor in susceptibility to SLE in several populations [8,10,12]. Interleukin-1 receptor antagonist (IL-1Ra) is a naturally occurring competitive inhibitor of IL-1. The IL-1Ra gene contains a polymorphism in intron 2 consisting of a variable number of copies of an 86-base-pair repeat sequence (two, three, four, five, or six copies) [13]. An association has been found between the IL-1Ra 2 allele and SLE [13,14]. Multiple genes are involved in the development of SLE, and the relative importance of these genes may vary between populations and with environmental exposure. We investigated common variant alleles involved in the immune response, immune complex clearance, and regulation of inflammation, with the hypothesis that combinations of polymorphic candidate genes could have synergistic effects on disease susceptibility. Therefore, we have analysed polymorphisms in the genes HLA DR, HLA DQ, C4A, FcγRIIa, FcγRIIIa, MBL, and IL-1Ra and their association with the development of SLE.

## Materials and methods

### Patients

The study population comprised 124 female and 14 male Caucasian SLE patients, and 200 blood donors (100 men, 100 women) were used as controls. One hundred thirty-eight patients fulfilled four or more criteria of the American College of Rheumatology (ACR) classification for SLE [15]. Five patients with a clinical SLE diagnosis were included in the study even though they fulfilled only three ACR classification criteria; these five patients had multisystemic disease with an immunologic disorder, i.e. presence of anitnuclear antibodies and symptoms characteristic of SLE such as arthritis, photosensitivity, serositis, nephritis, thrombocytopenia, and leucopenia [16]. A breakdown of the ACR criteria is shown in Table 1. There were 129 families with a single case of SLE and 14 families in which multiple cases were recorded. However, from each multicase family, only the first family member with SLE diagnosis, the index case, was included in the statistical analysis. The mean age at diagnosis of the patients was 40 years (range 10–83) and the mean disease duration was 16 years (range 1–42). The mean Systemic Lupus International Collaborating Clinics/ACR-Damage Index score was 1.9 (range 0–9) [17]. The study was approved by the local ethics committee at Lund University.

### Genetic analyses

DNA was extracted by the salting-out method described by Miller and colleagues [18]. Analysis of genetic polymorphism was predominantly performed by polymerase chain reaction (PCR).

#### HLA

HLA DR and DQ alleles were determined with PCR (Olerup SSP™ DQ-DR SSP Combi Tray, Olerup SSP AB, Stockholm, Sweden). However, a minority of the patients had previously been typed with a lymphocytotoxicity test or by restriction fragment length polymorphism as described before [2]. C4A gene deletion was determined by PCR as described by Grant and colleagues [19], or in a few cases by analysis of restriction fragment length polymorphism and determination of MHC haplotypes [2]. With the presence of a DR3 allele together with a DQ2 and a C4AQ0 allele, due to C4A gene deletion, the subject was considered to have the haplotype HLA DR3-DQ2-C4AQ0, although family studies were not uniformly performed to confirm this assumption.

#### FcγRIIa gene polymorphism

The genetic polymorphism resulting in amino acid R or H in amino acid position 131 was determined as previously described [20].

#### Analysis of FcγRIIIa gene polymorphism

The analysis of the F/V polymorphism was performed essentially as previously described [21].

#### MBL gene polymorphism

Variants of MBL due to mutations at codon 52 (D), 54 (B), and 57 (C) in exon 1 of the MBL gene and promotor variants at position -550 (H/L) and -221 (X/Y) were determined by allele-specific PCR amplification, essentially as described before [9]. The wild-type structural allele is designated A, while 0 is a description of the mutant alleles B, C, and D. Based on previously described associations between MBL genotype and serum concentrations, which were confirmed in our 200 healthy controls, the MBL genotypes were divided into three groups. Group 1 (MBL-low) consisted of patients with two structural mutant alleles (0/0) or on one haplotype a structural mutant allele together with another haplotype containing an LX promoter and the wild-type structural allele (ALX/0). Group 2 (MBL-intermediate) consisted of patients with the promoters LX conferring low serum MBL on both haplotypes but with normal structural alleles (ALX/ALX), or, alternatively, haplotypes with one mutant and one wild-type structural allele with a non-LX promoter together with the wild-type allele. Group 3 (MBL-high) included patients with the A/A genotype and at least one non-LX promoter.

#### IL-1Ra gene polymorphism

Genetic polymorphism in the IL-1Ra gene was determined with a PCR essentially as previously described [13,22], although one primer was modified.

Primers: 5'-CTC AGC AAC ACT CCT AT-3'

5'-TTC CAC CAC ATG GAA C-3'

The amplified fragment size depends on the number of repeats (two repeats, designated allele 2; three, allele 4; four, allele 1; five, allele 3; six, allele 5).

### Statistics

Two group comparison tests were performed using the Fisher exact test. Comparisons between multiple groups were made using the χ^2 ^multiple comparison test. Significance was considered when *P *<0.05. Correction for multiple comparisons was not applied to the results, because the study design consisted in hypothesis testing. The presence of synergistic interaction between genetic variants was investigated by calculating relative excess risk due to interaction (RERI) [23].

## Results

**Figure 1:**
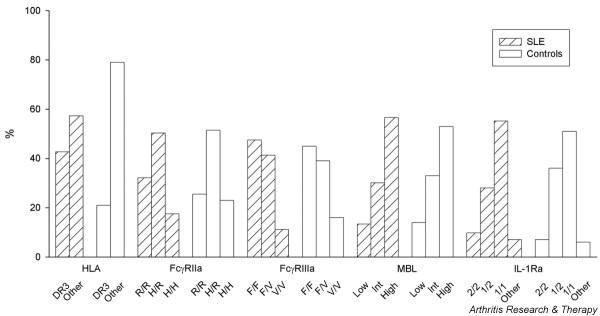
Distribution of genetic variants studied in 143 patients with SLE and 200 healthy blood donors. DR3 represents the haplotype DR3-DQ2-C4AQ0. F, phenylalanine; H, histidine; Int, intermediate; MBL, mannan-binding lectin; R, arginine; V, valine.

A strong association between the HLA DR3-DQ2-C4AQ0 haplotype and SLE was found, although this haplotype also was common among the controls. HLA DR2 was present in 50 of the 143 SLE patients and 72 of the 200 controls, while DR4 frequencies were 45/143 and 72/200, respectively. In the SLE group, HLA DQ2 was present in 80 of 143 cases, while DQ3 and DQ6 was recorded in 60 of 143 and 85 of 143 cases, respectively. The corresponding numbers in the control group were for DQ2, 73/200; for DQ3, 100/200; and for DQ6, 112/200. Other DR and DQ variants were less common. Ninety percent of the SLE patients with HLA DR3 displayed the haplotype DR3-DQ2-C4AQ0, compared with 86% of the controls. The frequencies of the FcγRIIa, FcγRIIIa, MBL, and IL-1Ra genotypes are displayed in Fig. 1. The FcγRIIa R/R, FcγRIIIa F/F, IL-1Ra 2/2, and MBL-low genotypes were not individually associated with SLE.

Additionally, the combination of genetic variants and susceptibility to SLE was tested (Table 2). HLA DR3-DQ2-C4AQ0 in combination with FcγRIIa R/R, FcγRIIIa F/F, or MBL-low was still associated with SLE but did not significantly increase the odds ratio (OR) in comparison with HLA DR3-DQ2-C4AQ0 alone. A combination of FcγRIIa R/R and IL-1Ra 2/2 yielded a strong association with SLE (OR 11.8), although the confidence interval was wide (1.5–95.4). Testing of RERI did not confirm the hypothesis that this interaction was synergistic (RERI 11.1, 95% CI -13.8 – 36.1, *P *= 0.38). A combined analysis of carriage rates for the R allele and the 2 allele (i.e. the patient should have at least one R allele and one 2 allele) was also performed, but no significant difference was detected between the SLE and the control group. No other combination displayed any association with SLE.

## Discussion

The increasing number of reports on polymorphic genes involved in susceptibility to SLE prompted us to investigate whether a combination of polymorphic candidate genes, tentatively thought to be involved in the pathogenesis of SLE, could further elucidate the genetic basis of the disease. In the present study we found that the combination of the FcγRIIa R/R genotype with the IL-1Ra 2/2 genotype was strongly associated with SLE. Although only a few of the patients had this particular genetic background, the results indicate that certain combinations of susceptibility genes can be of crucial importance. Furthermore, a strong association between the haplotype HLA DR3-DQ2-C4AQ0 and susceptibility to SLE was seen in this study, which is in concordance with the findings of previous studies [2,22,24,25]. The patients and controls studied were all from a homogeneous Caucasian population, although a possible bias exists in the fact that the controls used were blood donors, which principally include only healthy individuals, instead of age-matched controls from the normal population. The distributions of the polymorphic variants in the controls were in agreement with data published by others [13,26,27].

There have been ample studies on the association between FcγRIIa and SLE [24,28-30]. However, the results are somewhat conflicting regarding whether or not the R allele is associated with increased susceptibility to SLE in general or for SLE glomerulonephritis or other clinical manifestations of SLE. In our study, there was no association between either the R allele or the R/R genotype and susceptibility to SLE, with a glomerulonephritis frequency of 27%.

The MBL genotype did not seem to be involved in susceptibility to SLE in our Caucasian cohort. This differs from a finding of a recent meta-analysis in which MBL variant alleles were found to be associated with SLE [27]. Furthermore, in that study the conclusion was drawn that several studies are too small to detect an increased SLE susceptibility dependent on MBL risk alleles, which could also explain the lack of association in our study.

An increased carriage rate of the 2 allele of the IL-1Ra gene has been shown for SLE patients [13,14]. In our study, the 2/2 genotype in conjunction with the FcγRIIa R/R genotype was associated with SLE. This IL-1Ra genotype is associated with higher IL-1 beta concentrations as well as higher serum IL-1Ra levels [31,32]. Furthermore, immune complex binding to Fc receptors can influence the production of IL-1Ra [33], which provides a possibility for a pathogenetic mechanism concordant with the genetic interaction seen in our study. Analyses of disease phenotypes were beyond the scope of this study and will be addressed in future studies. However, there were no apparent associations between the various genotypes and clinical subsets of SLE. Because of the low number of patients included in the study, the results must be interpreted cautiously, and independent confirmation is needed.

## Conclusion

Our findings suggest that the combination of the FcγRIIa R/R and IL-1Ra 2/2 genotypes is associated with SLE in Caucasian patients, whereas individually these genotypes do not increase susceptibility to the disease. This finding illustrates that combinations of polymorphic genes may act in concert in the pathogenesis of SLE, a concept that may be instrumental in the analysis of the genetics of SLE as well as providing hypotheses for pathways in the pathogenesis of lupus.

## Competing interests

None declared.

## Author contributions

AJ was responsible for data analysis and interpretation and wrote the report.

AAB contributed to the data analysis and interpretation.

GS and LT were both responsible for the planning of the work and contributed to data analysis, interpretation, and write-up.

## Abbreviations

ACR = American College of Rheumatology; F = phenylalanine; FcγRIIa = Fcγ receptor IIa; FcγRIIIa = Fcγ receptor IIIa; H = histidine; IL-1Ra = interleukin-1 receptor antagonist; MBL = mannan-binding lectin; MBL-low/-intermediate/-high = MBL genotype conferring a low/intermediate/high serum concentration of MBL; MHC = major histocompatibility complex; OR = odds ratio; PCR = polymerase chain reaction; R = arginine; RERI = relative excess risk due to interaction; SLE = systemic lupus erythematosus; V = valine.

## Figures and Tables

**Table 1:** Distribution of American College of Rheumatology (ACR) classification criteria in 143 patients with SLE

ACR criterion	Patients	
	
	No.	%
Malar rash	79	55
Discoid rash	55	38
Photosensitivity	102	71
Oral ulcers	38	27
Arthritis	118	83
Serositis	76	53
Renal disorder	38	27
Neurologic disorder	12	8
Hematologic disorder	73	51
Immunologic disorder	103	76
Antinuclear antibody	143	100

**Table 2:** Comparisons of genetic variants in 143 patients with SLE and 200 healthy blood donors

Genetic variant	Patients		Controls				
					
	No.	(%)	No.	(%)	*P*^a^	OR^a^	95% CI^a^
HLA DR3-DQ2-C4AQ0	61	(43)	42	(21)	**<0.0001**	**2.8**	**1.7–4.5**
FcγRIIa R/R	46	(32)	51	(26)	0.18	1.4	0.86–2.2
FcγRIIIa F/F	68	(48)	90	(45)	0.66	1.1	0.72–1.7
MBL-low	19	(13)	28	(14)	0.88	0.94	0.50–1.8
IL-1 Ra 2/2	14	(9.8)	14	(7.0)	0.42	1.4	0.66–3.1
HLA DR3-DQ2-C4AQ0 / FcγRIIa R/R	20	(14)	7	(3.5)	**0.0005**	**4.5**	**1.8–10.9**
HLA DR3-DQ2-C4AQ0 / FcγRIIIa F/F	29	(20)	19	(9.5)	**0.007**	**2.4**	**1.3–4.5**
HLA DR3-DQ2-C4AQ0 / MBL-low	11	(7.7)	5	(2.5)	**0.04**	**3.3**	**1.1–9.6**
HLA DR3-DQ2-C4AQ0 / IL-1 Ra 2/2	4	(2.8)	3	(1.5)	0.46	1.9	0.42–8.6
FcγRIIa R/R / FcγRIIIa F/F	31	(22)	32	(16)	0.20	1.5	0.84–2.5
FcγRIIa R/R / MBL-low	3	(2.1)	6	(3.0)	0.74	0.69	0.17–2.8
FcγRIIa R/R / IL-1 Ra 2/2	8	(5.6)	1	(0.5)	**0.005**	**11.8**	**1.5–95.4**
FcγRIIIa F/F / MBL-low	8	(5.6)	12	(6.0)	1.0	0.92	0.37–2.3
FcγRIIIa F/F / IL-1 Ra 2/2	8	(5.6)	4	(2.0)	0.13	2.9	0.86–9.8
MBL-low / IL-1 Ra 2/2	1	(0.7)	0	(0.0)	0.42	4.2	0.17–104

^a^Bold type indicates statistical significance (*P *<0.05); CI, confidence interval; F, phenylalanine; MBL, mannan-binding lectin; MBL-low, MBL genotype conferring a low serum concentration of MBL; OR, odds ratio; R, arginine.
